# Natural Peptides in Drug Discovery Targeting Acetylcholinesterase

**DOI:** 10.3390/molecules23092344

**Published:** 2018-09-13

**Authors:** Vivitri Prasasty, Muhammad Radifar, Enade Istyastono

**Affiliations:** 1 Faculty of Biotechnology, Atma Jaya Catholic University of Indonesia, Jakarta 12930, Indonesia; vivitri.dewi@atmajaya.ac.id; 2 Department of Medical Laboratory Technology, Academy of Health Sciences Guna Bangsa, Condongcatur, Depok, Sleman, Yogyakarta 55283, Indonesia; radifar@gunabangsa.ac.id; 3 Faculty of Pharmacy, Sanata Dharma University, Paingan, Maguwoharjo, Depok, Sleman, Yogyakarta 55282, Indonesia

**Keywords:** natural peptides, acetylcholinesterase, drug discovery, inhibitor, Alzheimer’s disease

## Abstract

Acetylcholinesterase-inhibitory peptide has gained much importance since it can inhibit acetylcholinesterase (AChE) and increase the availability of acetylcholine in cholinergic synapses, enhancing cholinergic transmission in pharmacological treatment of Alzheimer’s disease (AD). Natural peptides have received considerable attention as biologically important substances as a source of AChE inhibitors. These natural peptides have high potential pharmaceutical and medicinal values due to their bioactivities as neuroprotective and neurodegenerative treatment activities. These peptides have attracted great interest in the pharmaceutical industries, in order to design potential peptides for use in the prophylactic and therapy purposes. Some natural peptides and their derivatives have high commercial values and have succeeded in reaching the pharmaceutical market. A large number of peptides are already in preclinical and clinical pipelines for treatment of various diseases. This review highlights the recent researches on the various natural peptides and future prospects for AD management.

## 1. Introduction

Acetylcholinesterase (AChE) (E.С.3.1.1.7) is a serine hydrolase which catalyzes the hydrolysis of neurotransmitter acetylcholine into choline and acetic acid [1,2]. According to cholinergic neurotransmission, acetylcholinesterase (AChE) inhibition would increase the levels of acetylcholine in the brain, thus improving cholinergic synapses in Alzheimer’s disease (AD) patients [3,4,5,6]. The impairment of cognitive functions and the behavioral disruptions that affect patients with AD are associated with deficiencies in cortical excitability, particularly cholinergic neurotransmission. Disturbances of acetylcholine metabolism have been detected in the AD patient brains with higher levels of AChE expression in decomposition of acetylcholine and deterioration in activity of choline acetyltransferase (ChAT), an enzyme which is responsible for the synthesis of acetylcholine [7,8,9].

AD is a progressive neurodegenerative disorder with complex multifactorial pathogenesis, memory impairment, and damaged cognitive skills, including attention, spatial orientation, judgment, language disorder, motoric problem, and loss in healthy aging [10,11,12]. The main hallmark of AD patient brain is characterized by extracellular senile plaques containing amyloid-β peptides (Aβ) deposits, neurofibrillary tangles, progressive synapse loss, and severe oxidative stress [13,14]. Aβ-induced oxidative stress is triggered by protein oxidation, lipid peroxidation, free radical formation, neuronal cell death via apoptotic pathway, DNA oxidation leading to DNA damage both in mitochondria and nucleus [15,16,17,18]. The most common form of AD syndrome is dementia. Dementia is a clinical syndrome characterized by progressive decline in cognitive function as well as behavioral disorders beyond the normal aging [19,20]. It is estimated that AD represents approximately 60–80% of all dementia cases [21,22]. Dementia mainly affects elderly and it is presumed that, by 2050, more than 115 million people suffer dementia, which majority contributed by AD [23]. In fact, most of the AD symptoms were related to loss of cholinergic function in the basal forebrain, the discovery of AChE inhibition is important for the treatment of AD [24].

One of the best approach in devastating AD is based on accelerating decomposition of available of acetylcholine which mainly improves the pathological symptom [25,26]. Inhibition of AChE is a promising strategy to develop novel and causal therapeutics in AD treatment [27,28]. AChE inhibitors are one of the most intensively probed categories of compounds in seeking an effective treatment of AD [29,30]. Another member of the cholinesterase (ChE) family is butyrylcholinesterase (BChE), which is also considered as promising target for AD drug discovery since the BChE activity increases in the late stages of AD. A previous study reported that BChE inhibitors could act in a similar way as well as AChE inhibitors, in particular to slow down cognitive decline [31]. AD drugs used recently as AChE inhibitors include tacrine, rivastigmine, galantamine, metrifonate, and donepezil [32,33]. However, these drugs may lead to injurious gastrointestinal side effects such as nausea, vomiting, diarrhea, and anorexia [33,34,35,36]. Currently, there is no cure for AD and these drugs only can temporarily improve symptoms or slow the progression of AD [37,38]. Thus, current approaches to the treatment of AD targeting AChE need be developed.

The other safer sources that can be used as AChE inhibitors are obtained from natural peptides. These peptides can be found in various sources, such as protein hydrolysates. Protein hydrolysate is a good source of nutrients for health due to it contains a high natural peptide compounds. Protein hydrolysates are well-known as mixtures of polypeptides, oligopeptides, and amino acids that are manufactured from protein sources using partial hydrolysis [39,40,41]. The advantages of consuming protein hydrolysate containing natural peptides due to its small molecular size thus easily absorbed in the digestive tract and quickly delivered into the bloodstream reaching muscle tissue, immune cells, internal organs, and brain cells. Protein hydrolysate is also known as food protein-derived peptides. Animal experiments prove that protein-derived peptides in the form of bovine casein-derived peptide (CH-3) are capable of preventing cognitive decline in Alzheimer’s disease of mice model with a decreasing of inflammation and oxidative stress. The suppressive effect of CH-3 is mainly due to the tripeptide: methionine-lysine-proline (MKP). Hence, this study suggests that CH-3 has therapeutic potential for preventing cognitive impairment in AD [42]. Moreover, a previous study also reported that 2,5-Diketopiperazine (DKP), also known as cyclic dipeptides which contain cyclo(-Phe-Phe), have received considerable attention as biologically important molecules as a source of AChE inhibitory peptide [43,44]. DKP can be synthesized from the N-terminus of amino acid residues of a natural linear peptide and can be found in whey protein hydrolysates.

There is an abundance of natural peptides (NPs) which are known as specific protein fragments and the majority have positive impacts on neurodegenerative preventives and treatments [45,46,47,48,49]. To date, more than 1500 different NPs have been studied as functional biomolecules including neuropeptides in a database called ‘Biopep’ [50,51]. NPs display drug-like or neurodegenerative treatment activities as neuropeptides have been reported in many previous studies [52,53,54,55]. Most natural neuropeptides were isolated using bioassays based on physiological impacts as well as light responses, spontaneous potentials responsible for the rhythmic contraction of the gut, motor patterns, or heartbeat frequency changes [56,57,58]. Neuropeptides noticed in a fraction form as biologically functional molecules, have been identified and isolated from various natural sources, and their activities were investigated in many medical and pharmaceutical purposes [59,60,61,62]. Here, we present a review which is mainly concerned with NPs targeting AChE in the context of different natural source matrices.

## 2. Neuropeptides from Plant Sources

Plants are rich sources of pharmacologically bioactive agents and some studies of Portugal’s medicinal plants showed great results for AChE inhibition with the species *Melissa officinalis*, *Mentha suaveolens*, *Laurus nobilis*, *Hypericum undulatum*, and *Sanguisorba minor* [63,64]. These plants were used in traditional medicines, in particular, for the treatment of nerve dysfunction or related problems over three decades [64]. Recently, hemp seed peptides targeting AChE-inhibitory enzymatic hydrolysates were obtained using six distinct proteases (pepsin, pepsin-pancreatin, alcalase, papain, flavourzyme, and thermoase) at a range of concentrations (1–4%). The activity of hemp seed peptides showed that about 1% pepsin hemp seed peptides had high activity of AChE inhibition with IC_50_ value was ~6 × 10^−3^ mg/mL compared to IC_50_ value was within 8–11.6 × 10^−3^ mg/mL from other enzymatic hemp seed peptides. The analysis of mass spectrometry exhibited that most of the peptide sizes in all the hydrolysate forms were less than 1000 Da: 244–1009 Da for the pepsin hemp seed peptides, 246–758 Da for the papain hemp seed peptides, and 246–607 Da for the alcalase hemp seed peptides. The pepsin hemp seed peptides showed higher AChE-inhibitory effects which might be based on the enhanced synergistic effects from a longer peptide size range when compared to the alcalase and papain hemp seed peptides which had a smaller size range [53].

A more recent study following Tsai et al. [65] was the identification of the polysaccharide–peptide complex in AChE inhibitory activities from the mushroom, *Cordyceps militaris* (CPSP) and characterization of AChE inhibitory properties. The covalent bond of polysaccharide–peptide complexes are attributed to O-glycosidic linkages in CPSP-F1 and N-glycosidic linkages in CPSP-F2 and CPSP-F3. The three polymers of CPSP-F1, CPSP-F2, and CPSP-F3 were extracted and separated by ultrasound-assisted extraction and diethylaminoethanol (DEAE)–Sepharose CL-6B column chromatography. Polysaccharide–peptide complexes were identified by DEAE-Sepharose CL-6B column chromatography followed by high-performance gel-filtration chromatography. The results showed that CPSP-F1 and CPSP-F2 had half maximal inhibitory concentrations of 32.2 ± 0.2 mg/mL and 5.3 ± 0.0 mg/mL, respectively.

Meanwhile, the fruit peptides of *Ziziphus jujuba*, known as the jujube or Chinese date, a species of Ziziphus in the buckthorn family Rhamnaceae, are being consumed worldwide due to their health benefits, as both food and herbal medicine. As herbal medicine, one of the main functions of jujube is to relax the brain by calming down the mind and improving sleep quality. Jujube is known to possess neuroprotective activities, including protecting neuronal cells against neurotoxin stress, stimulating neuronal differentiation, increasing expression of neurotrophic factors, and promoting memory and learning [66]. A study showed that the *Zizyphus jujube* fruit exerts neurobiological effects, especially antioxidant and anticholinesterase activities [67]. The fruit peptide of jujube was introduced as a new peptide and named Snakin-Z, and consists of 31 amino acids with a sequence: CARLNCVPKGTSGNTETCPCYASLHSCRKYG. The Snakin-Z activity showed considerable inhibition against AChE. The half maximal inhibitory concentration (IC_50_) value of Snakin-Z against AChE was 0.58 ± 0.08 mg/mL. This peptide has 80% enzyme inhibitory activity on AChE at 1.5 mg/mL. The Snakin-Z also had high antioxidant activity (IC_50_ = 0.75 ± 0.09 mg/mL). Thus, it is suggested that Snakin-Z might be beneficial for AD treatment [68]. Another beneficial part of the jujube is its seed. Recent findings indicate that the seed of the jujube ameliorates an Aβ-induced synaptic long-term potentiation (LTP) deficit in the hippocampal tissue through BDNF/TrkB signaling. This phenomenon is induced by a regulatory effect of jujube seed on the post-translation modification of the brain-derived neurotrophic factor (BDNF) [69].

The fermentation of some edible plants are also popular in the discovery of new AChE inhibitors. A traditional Chinese salt-fermented soybean, known as commercial Douchi, has been determined to have acetylcholinesterase (AChE) inhibitory activity. The results indicated that nineteen samples of commercial Douchi showed various extents of AChE inhibitory activities. The IC_50_ value of AChE inhibitory activities of Douchi extracts ranged from 0.040 to 2.319 mg/mL. Douchi extracts using Aspergillus exhibited significantly higher AChE inhibitory activity than using Mucor and Bacillus [70]. The other fermentation based product is sufu (fermented tofu), a traditional Chinese food, that has been manufactured by fermenting soybean (yellow soybean & black soybean). Sufu was reported as having AChE inhibitory activities. The bioactivities of anti-AChE from 15 brands of commercial sufu samples were taken from various regions in China, and self-produced sufu produced by fermentation with *Actinomucor elegans* 3.118, was successfully determined. The results indicated that sufu extract performed significant inhibition activity toward AChE in vitro. It proved that sufu no. 5 had the strongest inhibitory activity (IC_50_ = 0.191 mg/mL), while the prefermented sufu showed the highest AChE inhibitory activity during sufu production. According to this result, it might be possible that Chinese sufu could be considered in the treatment of Alzheimer’s disease patients [71]. The source of fermented milks cultured with various probiotic strains could improve amyloid precursor protein (APP) metabolism in Alzheimer’s disease. Fermented milk cultured with *Lactobacillus helveticus* IDCC 3801 induced a strong decrease in the incidence of Alzheimer’s disease but also ameliorated scopolamine-induced amnesia. This also suggests that the extract from fermented milk with *L. helveticus* IDCC 3801 may improve APP metabolism and memory deficit [72].

Rice bran is one of bioactive food-derived peptides source and possesses the capability to enhance positive health by decreasing chronic complication risks including obesity and aging diseases such as AD. The high demand of rice bran in the global food market has supported extensive research on natural peptides that are used both in prevention and treatment of AD. Fractions of peptides from rice bran have been identified and characterized for potential inhibitory activities against AD. This peptide showed nearly 45% reduction in cell cytotoxicity on amyloid-induced neuronal cells. Hence, rice bran peptides could possibly to be used as natural nutraceuticals to help with the management of AD [73,74].

## 3. Neuropeptides from Marine Sources

Approximately 70% of the Earth’s surface is covered by saltwater oceans which occupy more than 90% of the biosphere [75,76]. Oceans are a rich source of bioactive compounds with diverse structures and functions as well as potential therapeutic agents [75,76]. In recent years, many bioactive compounds have been extracted from various marine animals like sea snails, sponges, soft corals, marine fungus, marine algae, and marine microbes [77,78,79,80,81]. The exploration of new metabolites from marine organisms has become popular, and approximately 10,000 bioactive compounds have been successfully isolated including polysaccharides, enzymes/protein, minerals, vitamins, polyunsaturated fatty acids (PUFA), alkaloids, phenols, terpenoids, and natural peptides [82,83,84,85,86,87,88,89,90,91]. Natural peptides generally have 3 to 20 amino acid residues, with their functions dependent on the composition and sequence length of amino acids. Natural peptides with a low molecular weight have an escalated feasibility of permeability through the gut wall without any disruption, hence employing their biological and functional activities [92,93].

Natural peptides were first discovered and isolated from marine organisms in the 1960s. After the discovery of neurotoxins, studies on marine peptides were done, vastly in the 1980s [62]. Approximately more than 2000 natural peptides have been isolated, representing 1/10 of the total bioactive compounds obtained from marine organisms [94]. Thus, there is abundant amount of published articles relating to marine-derived peptide isolation. Since then, the explorations of marine neuropeptides have continued with the purpose to assure their application in treatment or prevention of neurodegenerative diseases. 

### 3.1. Cone Snail Conotoxins

Conotoxins are neuropeptides isolated from venom of sea snail or cone snails (*Conus* sp.) which normally consist of 10 to 30 amino acid (A.A.) residues with one or more disulfide bonds. Conotoxins have a variety of AA compositions and reaction mechanisms, but most of them have not been determined. The majority of conotoxins modulate the activity of ion channels. However, some conotoxins are also capable of inhibiting cholinesterase. Over the last few decades, conotoxins have been the subject of pharmacological interest. The venom of ω-conotoxin from marine cone snails has been proven to be a valuable device in neuroscience, employing a key role in the the identification and characterization of Voltage-dependent calcium channel (VGCC) subtypes [95,96].

Other evidence of conotoxin mechanisms in the inhibition of cholinesterases can be found in insect and mammalian nervous systems, in particular, presynaptic muscarinic receptors on the liberation of acetylcholine from striatal cholinergic neurons were investigated. The electrically stimulated release of [3*H*]-acetylcholine has been examined from rat striatal slices and its inhibition by carbachol are affected by specific inhibitors of voltage-operated calcium channels of the N-type by ω-conotoxin GVIA. The generated release of [3*H*]-acetylcholine was decreased by ω-conotoxin GVIA, indicating that the N-type channel was involved in the release of some neurotransmitters. The N-type channels were responsible for approximately two thirds of neurotransmitters release. The release was >97% blocked by ω-toxins. In the imaging experiment on brain slices, where cholinesterases had been inhibited by paraoxon, the inhibition of [3*H*]-acetylcholine release by endogenous acetylcholine accumulated in the tissue could be exhibited by the escalation of the release after atropine addition. The inhibition was high in slices with functional N-type channels. Thus, it was concluded that the N-type calcium channel may contribute to the stimulation of acetylcholine release in rat striatum [97].

In a recent study, Minic et al. showed that the potential of venom toxins for the inhibition of protein receptors in the human body was implicated in the pathophysiology of AD. AChE has been identified as a promising target for treatment of cognitive impairments associated with Alzheimer’s by applying its enzyme inhibitors. A conotoxin has been reported playing a role as an effective inhibitor of acetylcholinesterase. The in silico study of a certain conotoxin has been designed with acetylcholinesterase, and may open aspects in binding affinity and specificity improvement. Docking studies were performed to structurally determine the binding interactions involved in the binding pocket of venom toxins with acetylcholinesterase occupied by each toxin in complex with acetylcholinesterase. The binding mode between each toxin and AChE was similar to conotoxin and AChE. Hence, it is a great prospect to investigate conotoxins as potential reversible and irreversible inhibitors of acetylcholinesterase for the treatment of the cognitive disorder of Alzheimer’s disease [98].

### 3.2. AChEIs from Sponges

Previous studies reported that aqueous and organic extracts of tropical marine sponges have biological activities including sponge peptides towards AChE. Approximately 66 extracts were obtained and screened from 35 marine sponge species from the Caribbean Sea (Curaçao) and from 8 marine sponge species from the Great Barrier Reef (Lizard Island). The extracts were dissolved in aqueous and organic solvents then tested for anti-AChE activities. The most interesting findings were acquired from extracts of *Pandaros acanthifolium*, *Neofibularia nolitangere*, *Verongula rigida*, *Topsentia ophiraphidites*, and *Ircinia felix*. The inhibition activities against AChE from some sponges were generally found to be weak. However, the methanolic extract of *T. ophiraphidites* showed stronger anti-AChE activity compared to other sponge extracts [99].

Furthermore, the assessment activities of acetylcholinesterase inhibitors (AChEIs) of 134 extracts obtained from 45 species of marine sponges have been reported by Beedessee and coworkers [100]. Microplate and Thin-layer chromatography (TLC) assays had revealed potent activities of acetylcholinsterase inhibitors in two ethyl acetate sponge extracts: *Pericharax heteroraphis* and *Amphimedon navalis*. Further investigated extracts were found to possess inhibitory kinetics by exhibiting mixed competitive/noncompetitive inhibition. However, the pure peptides, such as AChEIs from marine sponges, are still difficult to explore. Thus, the potential of marine sponges as a source of AChEIs are essential in future, particularly the purification and characterization of solitary bioactive compounds for their therapeutic applications in combating neurodegenerative diseases. 

Turk et al. reported that approximately 33 deep-sea Antarctic marine sponges were successfully screened in ethanolic extracts and they showed strong inhibition toward acetylcholinesterase. The most eminent AChE activities were associated with the extracts from sponges belonging to the genus *Latrunculia*. While most of the AChE inhibition activities associate with known secondary metabolites, some other immensely strong acetylcholinesterase inhibitors still associate to the unknown compounds [101].

### 3.3. Marine Bacterial Peptides

A huge variety of bioactive compounds can be extracted from marine bacteria. This means that the field of marine bioactive compounds, such as peptides, is continuously expanding. Recent advances in biological techniques enable a high level interest in natural peptides being developed. An overview of the discovery of new marine bacterial peptides with potential biological activities against AChE has been widely explored.

Bacterial associates of marine soft corals and sponges with anticholinesterase activities were extensively reported in the previous studies. Approximately 887 marine bacteria were screened in a microplate-based assay for the potential inhibitions towards acetylcholinesterase, and around 140 marine bacteria (15.8%) were found to inhibit acetylcholinesterase found in the electric eel enzyme. The majority of the active bacterial isolates were associated bacteria with soft corals and marine sponges. The maximum inhibition was found about 54% by a bacterial strain M18SP4P, isolated from the marine sponge *Fasciospongia cavernosa*. Based on its phenotypic properties and 16S rDNA sequencing, the strain was identified as *Bacillus subtilis*. This result showed that acetylcholinesterase inhibitors are quite common found in marine bacteria [102].

Fengwu et al. [103] reported that isolated endophytic bacteria which were screened from oysters showed acetylcholinesterase inhibitory activity. Out of eight strains endophytic bacteria separated from oyster, one of them had AChE inhibitory activity, which was identified as *Bacillus subtilis*, the inhibition rate was 22.4%, and it increased 3.3-fold at 37 °C for 78 h incubation.

### 3.4. Marine Fungus Peptides

Marine fungi, particularly those associated with marine animals and plants, appear to be a uniquely rich resource for secondary metabolites, such as antibiotic, antitumor, antiinflammatory, antidiabetes, and anti-acetycholinesterase agents [104,105,106,107]. A study conducted by Wu et al. [107] reported that Talaromycesone A, talaroxanthenone, and AS-186c were isolated from the culture broth and mycelia of a marine fungus *Talaromyces* sp. strain LF458, displayed potent acetylcholinesterase inhibitory activities with IC_50_ values were 0.00749, 0.00161, and 0.0026 M, respectively. All isolated compounds were subjected to bioactivity assays.

## 4. Neuropeptides from Terrestrial Venomous Animal

Venomous animals are widely spread all over the globe. Several terrestrial vertebrates (reptiles, birds, insects, and mammals) are also venomous [108,109]. Venomous animals permanently or periodically exploit self-generated substances for self-defense or capture prey that are toxic to other species and cause death, even in small doses [110,111]. Several venomous animals have special venom glands to produce their venom, while others contain toxic substances in various body parts. A number of animals have a specialized organ used for introducing their venom into prey’s body. Major arthropods (scorpions, bees, and wasps) have multicellular glands attached to the stinging apparatus. In most terrestrial venomous, animals such as spiders, snakes, and millipedes, their venom glands are connected to their mouthparts and the venom is spurted into the body of prey through prick or bite. The most studied of the venomous terrestrial animals are snakes, spiders, and scorpions [112,113]. It is known that animal venoms are complex mixtures with compositions depending on the species [114].

Bee venom obtained from stepwise fractionation with methanol, acetone, polyacrylamide, Sephadex gels, and anion exchangers has been reported to contain the polypeptides minimine and melittin. One of these polypeptides has a molecular weight of around 6000 Da. This polypeptide was successfully inhibited growth of third-instar larvae with a LD_50_ dose value of approximately 0.005 μg. On the other hand, these polypeptides were also reported to have in vitro anti-acetylcholinesterase activity [115].

The Taiwan snake (*Bungarus multicinctus*) possesses venom called α-bungarotoxin, a three-finger α-neurotoxin that has action against the acetylcholine receptor (AChR). The remarkable three-finger toxin fold has maximally thrived to use a distinct combination of functional groups to generate an armor of certain targets of AChR subtypes [116]. Powerful inhibition of mammalian acetylcholinesterase was detected in the venom of the green mamba snake, *Dendroaspis anyusticeps*. The compound responsible for inhibiting AChE was isolated and purified by gel filtration on Sephadex G-50, followed by ion exchange chromatography on Bio-Rex 70 and SP Sephadex C-25, respectively. These substances are known as polypeptides, named fasciculins [117].

## 5. Neuropeptides from Venomous Amphibian 

In general, amphibians have one or more efficacious neuropeptides in their dorsal skin secretions. The most common neuropeptide belongs to amphibians of the frog genera, called caerulein, with peptide sequence: [pEQDY(SO_3_)TGWMDF-NH_2_]. Caerulein has various biomedical activities at nanomolar concentrations. The penetration of caerulein is known to be able to affect smooth muscle contraction, reduce blood pressure, have gastrin-like activity, change satiety-related sedation, and thermoregulation. The Australian frogs from genus Litoria have the capability to modify caeruleins in different seasons. The Uperoleia genus of toadlets can produce a number of tachykinin- and bombesin-type neuropeptides, including uperolein [pEPDPNAFYGLM-NH2], while froglets of the genus Crinia can produce various disulfide-containing neuropeptides that cause smooth muscle contraction at very small concentrations (nanomolar size), such as assigniferin 1 peptide, RLCIPYIIPC-OH. These peptides can be used in the study of drug-induced pancreatitis as biomarkers of autophagy and pancreatic cell apoptosis; autophagy malfunction is now considered a major contributor to Alzheimer’s disease and other neurodegenerative disorders [118,119].

Amphibian defensive skin secretions are known to have a plethora of biologically-active peptides that are structural and functional analogs of vertebrate neuropeptides. Wang et al. reported that the amino acid sequences of two invertebrate neuropeptide analogs are IPPQFMRF amide (IF-8 amide) and EGDEDEFLRF amide (EF-10 amide) isolated from the defensive skin secretions of two different species of African hyperoliid frogs: *Kassina maculata* and *Phylictimantis verrucosus* [120]. These neuropeptides represent the C-terminal tetrapeptide amides of both novel peptides: –FMRF amide and –FLRF amide, reflecting the canonical structural motifs of the FMRF amide-related peptides (FaRPs) which are recognized as the ubiquitous invertebrate neuropeptide family. FaRPs show the potential targets of the defensive arsenal of amphibian tegumental secretions to invertebrate predators, and the possibility of more novel peptides from amphibians may represent more potent vertebrate neuropeptides.

Siano et al. reported that the amphibian skin peptide found with sequence: TKPTLLGLPLGAGPAAGPGKR-NH_2_ possessed a significant AChE. The present work demonstrates that subtraction of the size of the peptide origin could potentially form new compounds with significant cholinesterase inhibition activity [121].

## 6. Strategies for the Rational Design of Peptide as Therapeutic Leads

Peptide therapeutics are exclusively associated with lower production complexity compared with protein-based biopharmaceuticals. This is of course beneficial, considering the low cost of drug production, due to generally approaching those of small molecules. Therefore, peptides are in the sweet spot between small molecules and biopharmaceuticals in many ways. Natural peptides are frequently not immediately suitable for use as therapy because of intrinsic weaknesses, including poor chemical and physical stabilities, and a short plasma half-life. Some of these weaknesses have been successfully resolved through the conventional design of therapeutic peptides. Besides conventional natural peptide design, the wide range of peptide technologies has been presenting new opportunities and future directions within the peptide field. These include multifunctional peptides in cell penetration, as well as conjugation of peptide drugs and effective technologies focusing on selection routes of administration [122,123]. Peptides have thrived as highly potent signal transduction molecules, employing powerful physiological effects. Peptides are generally categorized by a relatively short turning plasma half-life, as well as physicochemical properties for their use in biomedical and pharmaceutical fields. Therefore, conventional rational design of peptide therapeutics has focused on rectifying techniques to relieve susceptibilities [123]. Several modified natural peptides with activity against acetylcholinesterase of Alzheimer’s disease are shown on Table 1.

Peptide toxicity has been evaluated mostly by investigating the hemolysis activity toward human red blood cells [124]. The Melittin peptide is known to be high cytolytic in bioassays. Its action is beneficial to block the electrostatic interactions that most peptides need to interact with microbes [125]. Toxicity assays should be conducted under conditions mimicking the environment that the peptides will confront in the host cells [126]. In selected examples, the toxicity assays have been performed in various in vitro mammalian cell cultures and in vivo animal model viabilities in the presence of peptides [127,128]. Based on peptide stability, extracts obtained from different mammalian tissues or commercially available proteases are well fit to determine peptide susceptibility to molecular target degradation and may affect the peptide activities [129,130].

The decreasing number of approved drugs produced by the pharmaceutical industry, which is accompanied by the increasing expense of R&D, is causing a high demand for alternative approaches to increase pharmaceutical R&D productivity. This circumstance has supported a revival in peptide research as potential drug candidates. New synthetic strategies for confining metabolism and alternative routes of administration have appeared in recent years and produced a large number of peptide-based drugs that are now being marketed. Peptide-based drug discovery will be important for answering new therapeutic challenges [131].

### 6.1. Peptide Design by Chemical Synthesis

Since Bruce Merrifield invented the methods of solid-phase peptide synthesis (SPSS) in the early 1960s, an increasing number of research groups have focused on peptide synthesis. However, the classical step-by-step procedures for peptide synthesis still has limitations, such as decreasing final product purity after passing through several coupling steps. It is understandable that a challenging method for the activation of a peptide terminal group needs protecting groups, both t-butoxycarbonyl (Boc) and 9-fluorenylmethoxycarbonyl (Fmoc). Besides Boc and Fmoc, novel amino acid protecting groups and new techniques have been largely introduced to improve the quality and quantity of peptide products. The condensation of the peptide fragment method was a popular method for peptide production in the 1980s. In contrast, the racemization rate and overall reaction still have difficulties to be resolved. In overcoming these issues, Kent and coworkers introduced the peptide coupling revolution by utilizing the chemoselective reaction of unprotected peptide segments, namely native chemical ligation. Later on, a large number of studies have focused on the development of propitious ligating techniques including the well-known click reactions, ligation of peptide hydrazides, and ligation of 5-oxaproline with α-ketoacid-hydroxylamine [132,133,134].

Another promising strategy in rational peptide drug design is to improve the physicochemical properties of natural peptides, which often have a tendency to aggregate and sometimes poorly dissolve in water. As expected from chemical design strategies, the development of streamlined techniques other than chemical conversion were highly needed for eco-friendly peptide synthesis to avoid aggregation, such methods include the peculation of hydrophobic darns, which can be achieved by substituting or catalyzing N-methylation of the incorporated amino acids. If solubility issues arise in certain peptide drug candidates, the regular basis zone is on molecular charge allocation and the isoelectric point (pI) of the peptides, which are related to favorable pH and purification to obtain the desired final product [123].

The chemical optimization strategy of a therapeutic peptide is based on the structure–activity relationship and/or quantitative structure–activity relationship studies of newly synthesized peptide derivatives, with the objective to improve bioavailability, limit biodegradation, reduce elimination, and increase affinity or selectivity to its target or receptor. The main concern for the low oral bioavailability of peptide drugs are presystemic enzymatic degradation and poor penetration of the intestinal mucosa. According to Lipinski’s rule of five evaluations, some peptides may be poor candidates to shift from the gastrointestinal tract to the circulatory system due to their unfavorable physicochemical properties [135,136].

Many efforts have been done regarding enhancing drug delivery by designing drug substances as prodrugs to improve drug penetration via transcellular pathways to treat patients. The other alternative route goes through the paracellular pathway by modulating the intercellular junction protein clusters. It is presumed that this approach has a remarkable future prospect in oral delivery of large molecules such as peptides, proteins, and oligonucleotides. Moreover, this approach has the potential to deliver large molecules to the brain as diagnostic and therapeutic agents for brain diseases such as Alzheimer’s disease [137,138,139].

### 6.2. Peptide Design by Molecular Biological Approach 

Bioactive peptides can be acquired immediately from living organisms by the hydrolysis of proteins or by chemical synthesis and these approaches are not cost-effective. A biological expression system using genetic techniques based on a fusion technology would be a more efficient method for the production of bioactive peptides. However, the molecular biology research has led to the availability of amino acid sequence data determined by the cloning and cDNA sequencing of genes [140].

The production of natural neuropeptides in competent host cells has been studied by Rosenfeld and coworkers. They used alternative processing of the RNA transcribed from the appeared calcitonin gene to obtain a messenger RNA production in neural tissue distinct from that in parafollicular cells as called thyroid cells. The thyroid mRNA encodes a precursor to the hormone calcitonin whereas that in neural tissues generates a designed neuropeptide, referred to as calcitonin gene-related peptide (CGRP). This approach allows the application of recombinant DNA technology to analyze the complex neurobiological systems in the absence of previous structural and functional information from living organisms [141].

### 6.3. Peptide Product by Designed Enzymatic Degradation

Enzymatic hydrolysis of proteins is catalyzed by proteases, which cleave peptide bonds between two amino acids consuming a molecule of water per bond cleaved. Hence, the continuous cleavage of peptide bonds breaks down proteins into products of lower molecular weight such as peptones, peptides, and amino acids. Independently of the type of food protein, the enzymatic hydrolysis process commonly comprises the following stages. Grinding the raw material and homogenization in water (or buffer), temperature equilibration, and pH adjustment to the optimum values of the enzyme employed, followed by enzyme addition. Recently, ultrasonic assisted hydrolysis was evaluated with the purpose of facilitating the production of low molecular weight peptides. Upon completion of the reaction, the enzyme needs to be inactivated by heating or pH adjustment. Alternatively, continuous membrane reactor, where the enzyme is continuously recycled to the reaction tank, might also be used in order to stop the reaction and save enzyme costs.

Afterwards, the digested material, which contains the bioactive peptides, is separated from the precipitate and lipids by several methods: centrifugation or decantation, fractionated, and further stabilized by spray-drying. In utilizing an appropriate enzyme and having good control over processing conditions including pH, temperature, enzyme/protein ratio, and time, are critical points for the production of protein hydrolysates with good properties. Eventually, these process variables determine the extent of the hydrolysis reaction for a protein–enzyme system. This is normally indicated by the degree of hydrolysis (DH), which is defined as the percentage of peptide bonds cleaved. There are several methods to determine the DH such as pH-stat, trinitrobenzenesulfonic acid (TNBS), O-phthaldialdehyde (OPA), trichloroacetic acid soluble nitrogen (SN-TCA), and formol titration methods.

Several novel AChE-inhibitory peptides were discovered and their amino acid sequences were elucidated. Hemp seed proteins (HSPs) were enzymatically hydrolyzed and the released peptides were investigated as potential therapeutic agents. Membrane isolated HSPs (mHPI) were the most soluble with >60% solubility at pH 3–9 when compared to 27% solubility of isoelectric pH precipitated proteins (iHPI). However, iHPI formed emulsions with smaller oil droplet sizes (<1 µm) while mHPI formed bigger oil droplets. The iHPI was subjected to enzymatic hydrolysis using different concentrations (1–4%) of six proteases including pepsin, pancreatin, flavourzyme, thermoase, papain, and alcalase, to produce various HSP hydrolysates (HPHs). Some of HPHs amino acid sequences were identified to successfully produce and confirm the in vitro AChE-inhibitory activities. Subsequently, it could be used as a prospective model for future design that may serve as potent AD therapeutic agents [142].

## 7. Future Perspectives of Virtual Screening on Natural Peptides

In recent years, virtual screening has been used to assist the binding models suggestion between bioactive natural products with their molecular targets systematically [153,154]. A combination of pharmacophore modeling, virtual screening, and molecular docking studies provides information to identify and design AChE inhibitors with higher selectivity [155]. In a previous study, a retrospectively validated structure-based virtual screening (SBVS) protocol to identify potent AChE inhibitors has been developed [156]. The construction and the retrospective validation of the SBVS protocol [156] made use of the enhanced version of the database of useful decoys (DUD-E) [157]. The decoys in DUD-E were selected from the ZINC database [158,159]. Therefore, we were tempted to apply the validated SBVS protocol [156] to identify potent AChE inhibitors from natural products collected in the newest version of the ZINC database, ZINC15 [159].

In total, 190,090 natural products from ZINC15 were subjected in the screening campaign. Lipinski’s rule of five for drug likeness [160] was used as the first filter to discard nondruggable compounds in this campaign [161,162]. The filter resulted in 79,711 compounds that remained to be further screened virtually using the retrospectively validated SBVS protocol [156]. Only 23 compounds were identified as potent AChE inhibitors. Thence, the overall hit rate was 0.01%, which was considered as very low. Nevertheless, these identified potent compounds could serve as the pivotal compounds in the development of drug for AD [163,164,165] since the database for the retrospective validation which was compiled by Mysinger et al. [157] defined potent AChE inhibitors as inhibitors “with affinities (IC_50_, EC_50_, Ki, Kd, and log variants thereof) of 1 μM or better”. The following are the ZINC code [159] of the hit compounds: ZINC000253412911, ZINC000253411436, ZINC000095919360, ZINC000095486033, ZINC000085549645, ZINC000085626729, ZINC000085629900, ZINC000085772672, ZINC000085772673, ZINC000085641926, ZINC000085597045, ZINC000085595326, ZINC000014443683, ZINC000008791438, ZINC000008765420, ZINC000002123767, ZINC000002104243, ZINC000002159521, ZINC000071318273, ZINC000034074522, ZINC000049601562, ZINC000038993374 and ZINC000038661945. Notably, similar to peptides, nine out of the 23 hits contained an amide bond (Table 2). On the other hand, the attempts to screen all virtual 400 dipeptides and 8000 tripeptides by employing the same SBVS protocol resulted in zero hits. Therefore these nine compounds are of considerable and timely interest. Short molecular dynamics (MD) simulations of enzyme-inhibitor using NAMD [166] by employing the SBVS results of the compounds of interest as the starting points have been performed. These MD simulations were performed to prioritize the compounds and to analyze the plausible AChE-ligand binding interactions. The results indicate that compounds ZINC000002159521 and ZINC000038661945 are potential to be further tested since their minimum total energy were lower than the minimum total energy of AChE without ligand at MD simulations 0 to 200 ps (Table 2).

**Figure 1:**
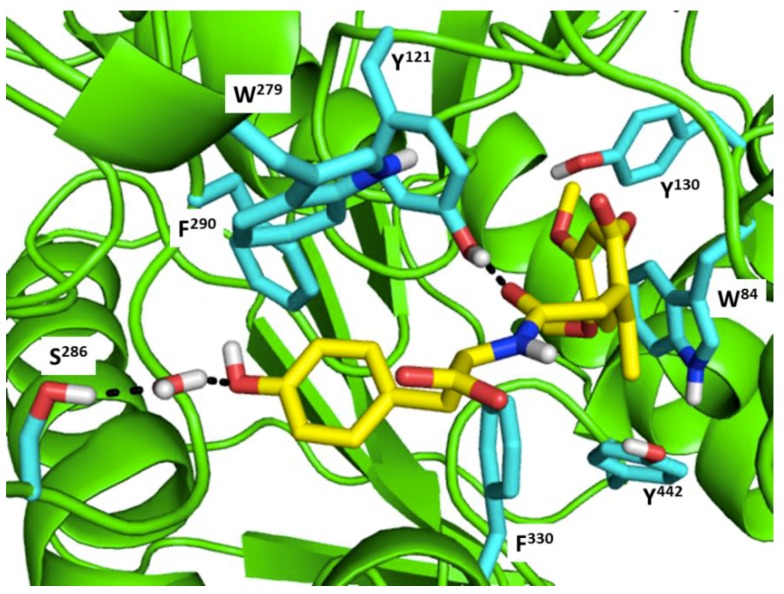
Compound ZINC000002159521 in the AChE binding pocket at its lowest total energy from the short MD simulations. The main chain of AChE is presented as a cartoon in green. The carbon atoms of the binding residues and ZINC000002159521 are presented as sticks in cyan and yellow, respectively. The oxygen and hydrogen atoms are presented as sticks in red and white, respectively. The hydrogen bonds are presented as black dashed lines.

Figure 1 shows the interactions of compound ZINC000002159521 at its lowest total energy from the MD simulations as a representative to examine the AChE-ligand binding interactions. The need of an aromatic moiety in the ligand to bind to AChE binding pocket [156] is clearly seen in Figure 1. The aromatic chromenone moiety from ZINC000002159521 is directly surrounded by aromatic residues in the AChE binding pocket, i.e.: W^84^, Y^130^, F^330^, and Y^442^. On the other subpocket, the phenol moiety from ZINC000002159521 is located adjacent to W^279^ and F^290^. Notably, the O carbonyl from the amide bond in ZINC000002159521 serves as hydrogen bond acceptor for Y^121^. This indicates the importance of amide bond for inhibitor to bind to AChE. Another identified hydrogen bond is the bond between O hydroxyl from the phenol moiety in ZINC000002159521 as hydrogen bond acceptor to S^286^ by employing a water molecule for bridging the bond. These insights from molecular modeling approaches could provide significant helps in the discovery and design novel AChE inhibitors.

## Figures and Tables

**Table 1:** Modified natural peptide analogs with activity against acetylcholinesterase of Alzheimer’s disease.

Peptide ID	Amino Acid Sequence	IC_50_ (M)	Source	Parental Compound	References
I_5_	Boc-VNLAG-OGal	34.46 × 10^−6^	Plant	galanthamine	[143,144]
KPF	KLPGF	1.09 × 10^−6^	Animal	Albumin	[145]
ARP	GMQGPAGSGWEEGSGSPPGVTPLFSP	n.a.	Animal	AChE-R	[146]
PF	EQRPR	n.a.	Plant	Protein hydrolysate	[73]
Colivelin	SALLRSIPAPAGASRLLLLTGEIDLP	10^−13^	Plant	ADNF C-terminally fused to AGA-(C8R)HNG17	[147]
AChE-peptide	AEFHRWSSYMVHWK	0.33	Human	AChE	[148,149]
T14	KAEFHRWSSYMVHWK	n.a.	Human	AChE	[150,151,152]
T15	NQFDHYSKQDRCSDL	n.a.	Human	AChE	[150,151]

ARP: Acetylcholinesterase Readthrough Peptide; PF: peptide fraction; n.a.: not available.

**Table 2:** Virtual screening hits containing amide bond and their minimum total energy values resulted from short MD simulations as potential acetylcholinesterase inhibitors (AChEIs).

Compounds	Total Energy Minimum * (kcal/mol)
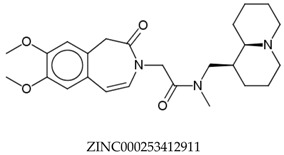	−172,400.8183
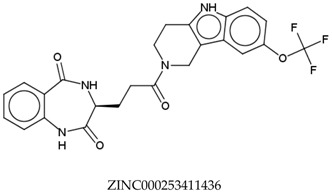	−172,400.7345
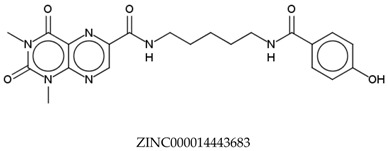	−172,547.1819
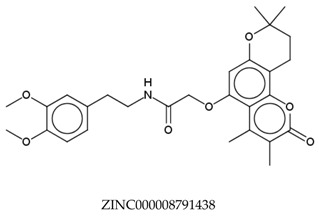	−172,021.4948
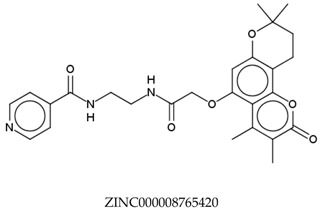	−172,053.5502
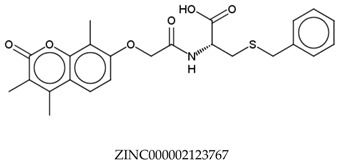	−172,517.7463
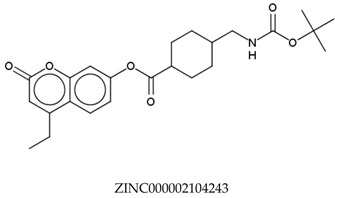	−172,451.0222
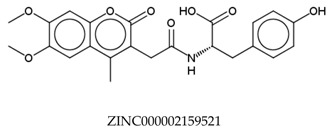	−172,754.0495
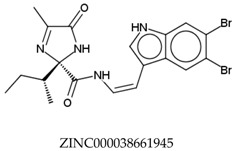	−173,190.7169

* The value for AChE without ligand is −172,691.7383 kcal/mol.
